# Poly[(μ_3_-biphenyl-3,4′-dicarboxyl­ato-κ^4^ *O* ^3^:*O* ^3′^:*O* ^4′^,*O* ^4′′^)(1*H*-imidazo[4,5-*f*][1,10]phenanthroline-κ^2^ *N* ^7^,*N* ^8^)manganese(II)]

**DOI:** 10.1107/S1600536810045587

**Published:** 2010-11-27

**Authors:** Fu-Ming Wang

**Affiliations:** a Department of Chemistry, Dezhou University, Shandong 253023, People’s Republic of China

## Abstract

In the title compound, [Mn(C_14_H_8_O_4_)(C_13_H_8_N_4_)]_*n*_, the Mn^II^ atom is six-coordinated in a distorted octa­hedral geometry by four O atoms from three different carboxyl­ate groups and two N atoms from one imidazo[4,5-*f*][1,10]phenanthroline mol­ecule. The organic ligands link inorganic Mn^II^ nodes, forming a zigzag chain along the *c* axis.

## Related literature

For the use of diphenic acid as an O-donor ligand in the design and synthesis of coordination polymers, see: Wang *et al.* (2006 ▶); Yin *et al.* (2005 ▶). The distortion of the diphenyl spacer about the central bond allows the carboxyl­ate ligand to link metal ions into helical chains or one dimensional chains, see: Guo *et al.* (2010 ▶). 
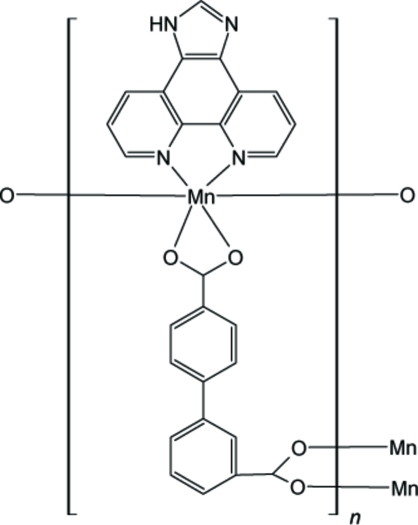


## Experimental

### 

#### Crystal data

[Mn(C_14_H_8_O_4_)(C_13_H_8_N_4_)]
*M* *_r_* = 515.38
Monoclinic, 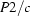

*a* = 8.0634 (13) Å
*b* = 11.705 (2) Å
*c* = 22.807 (4) Å
β = 94.307 (2)°
*V* = 2146.5 (6) Å^3^
*Z* = 4
Mo *K*α radiation
μ = 0.66 mm^−1^
*T* = 296 K
0.30 × 0.25 × 0.15 mm

#### Data collection

Bruker APEXII CCD area-detector diffractometer
Absorption correction: multi-scan (*SADABS*; Bruker, 2001 ▶) *T*_min_ = 0.915, *T*_max_ = 0.949
12211 measured reflections
3997 independent reflections
2211 reflections with *I* > 2σ(*I*)
*R*_int_ = 0.079

#### Refinement

*R*[*F* ^2^ > 2σ(*F* ^2^)] = 0.052
*wR*(*F* ^2^) = 0.107
*S* = 1.00
3997 reflections
325 parameters
H-atom parameters constrained
Δρ_max_ = 0.27 e Å^−3^
Δρ_min_ = −0.26 e Å^−3^

### 

Data collection: *APEX2* (Bruker, 2007 ▶); cell refinement: *SAINT* (Bruker, 2007 ▶); data reduction: *SAINT*; program(s) used to solve structure: *SIR97* (Altomare *et al.*, 1999) ▶; program(s) used to refine structure: *SHELXL97* (Sheldrick, 2008 ▶); molecular graphics: *SHELXTL* (Sheldrick, 2008 ▶); software used to prepare material for publication: *WinGX* (Farrugia, 1999 ▶).

## Supplementary Material

Crystal structure: contains datablocks global, I. DOI: 10.1107/S1600536810045587/jh2224sup1.cif

Structure factors: contains datablocks I. DOI: 10.1107/S1600536810045587/jh2224Isup2.hkl

Additional supplementary materials:  crystallographic information; 3D view; checkCIF report

## Figures and Tables

**Table:** 

Mn1—O4	2.106 (3)
Mn1—O3	2.124 (3)
Mn1—O2^i^	2.208 (3)
Mn1—N4	2.273 (3)
Mn1—N3	2.281 (3)
Mn1—O1^i^	2.313 (3)

**Table:** 

O4—Mn1—O3	97.67 (11)
O4—Mn1—O2^i^	89.59 (11)
O3—Mn1—O2^i^	98.36 (11)
O4—Mn1—N4	120.65 (11)
O3—Mn1—N4	94.32 (11)
O2^i^—Mn1—N4	145.23 (11)
O4—Mn1—N3	84.57 (11)
O3—Mn1—N3	165.21 (11)
O2^i^—Mn1—N3	96.26 (11)
N4—Mn1—N3	72.22 (11)
O4—Mn1—O1^i^	144.17 (10)
O3—Mn1—O1^i^	100.92 (10)
O2^i^—Mn1—O1^i^	57.65 (10)
N4—Mn1—O1^i^	88.24 (10)
N3—Mn1—O1^i^	85.10 (11)

Symmetry codes: (i) 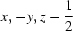; (ii) 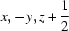.

**Fig. 1.**
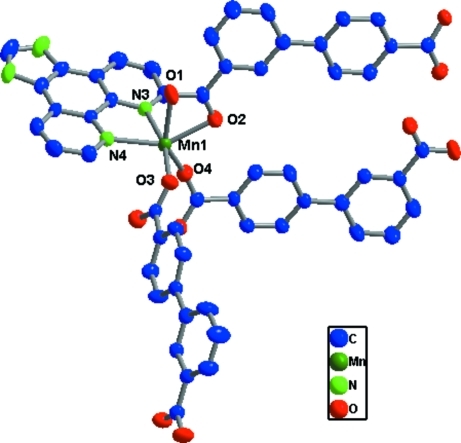
The coordination environments of manganese(II) atom. All hydrogen atoms are omitted for clarity.

**Fig. 2.**
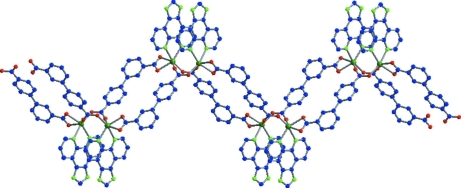
View of the one-dimensional zigzag chain running along c axis in compound

## supplementary crystallographic information

### Comment

Diphenic acid as O-donor ligand has received much more attention in the designed synthesis of coordination polymers (Wang, *et al.*, 2006; Yin,*et al.*, 2005). I select 3,4'-biphenyldicarboxylicacid as the ligand based on the following consideratons. First, the two functional carboxylate groups can adopt different coordination modes. Second, two phenyl rings are not coplanar with each other owing to the steric hindrance of carboxylate groups in coordinaton process. The distortion of diphenyl spacer about the central bond allows the carboxylate ligand to link metal ions into helical chains or one dimensional chains (Guo, *et al.*, 2010).

The title compound,(I), was synthesized by the hydrothermal reaction of 3,4'-biphenyldicarboxylic acid with imidazo[4,5-*f*][1,10]phenanthroline and manganese chloride terahydrate. The central Mn^II^ exhibits an octahedral geometry with N2O4 coordination sphere from three carboxylate ligands and one imidazo[4,5-*f*][1,10]phenanthroline ligand. The carboxylate groups act as m_3_-ligand with one carboxylate group bridging two Mn^II^ ions in a bis-monodetate fashion, and the other carboxylate group bridging Mn^II^ in a bidentate chelating mode. The dihedral angle two phenyl rings in carboxylate ligand is 9.33°. The carboxylate ligands link Mn^II^ nodes to form one-dimensional zigzag chain along *c* axis.

### Experimental

A mixture of MnCl_2_.4H_2_O (0.099 g, 0.5 mmol), 3,4'-biphenyldicarboxylic acid (0.121 g,0.5 mmol), NaOH (0.04 g, 1 mmol), imidazo[4,5-*f*][1,10]phenanthroline (0.110 g,0.5 mmol)and distillated water (15 ml) was heated to 433 K for 96 h in a 25 ml stainless steel reactor with a Teflon liner. Yellow block crystals were obtained with 52% yield on Mn basis.

### Refinement

Hydrogen atoms were included in calculated positions and refined with fixed thermal parameters riding on their parent atoms with C—H distances in the range of 0.93–0.98 Å.

### Crystal data

**Table:**

[Mn(C_14_H_8_O_4_)(C_13_H_8_N_4_)]	*F*(000) = 1052
*M**_r_* = 515.38	*D*_x_ = 1.595 Mg m^−^^3^
Monoclinic, *P*2/*c*	Mo *K*α radiation, λ = 0.71073 Å
*a* = 8.0634 (13) Å	Cell parameters from 1052 reflections
*b* = 11.705 (2) Å	θ = 2.5–19.6°
*c* = 22.807 (4) Å	µ = 0.66 mm^−^^1^
β = 94.307 (2)°	*T* = 296 K
*V* = 2146.5 (6) Å^3^	Block, yellow
*Z* = 4	0.30 × 0.25 × 0.15 mm

### Data collection

**Table:**

Bruker APEXII CCD area-detector diffractometer	3997 independent reflections
Radiation source: fine-focus sealed tube	2211 reflections with *I* > 2σ(*I*)
graphite	*R*_int_ = 0.079
φ and ω scans	θ_max_ = 25.5°, θ_min_ = 2.5°
Absorption correction: multi-scan (*SADABS*; Bruker, 2001)	*h* = −9→9
*T*_min_ = 0.915, *T*_max_ = 0.949	*k* = −14→13
12211 measured reflections	*l* = −27→27

### Refinement

**Table:**

Refinement on *F*^2^	Primary atom site location: structure-invariant direct methods
Least-squares matrix: full	Secondary atom site location: difference Fourier map
*R*[*F*^2^ > 2σ(*F*^2^)] = 0.052	Hydrogen site location: inferred from neighbouring sites
*wR*(*F*^2^) = 0.107	H-atom parameters constrained
*S* = 1.00	*w* = 1/[σ^2^(*F*_o_^2^) + (0.026*P*)^2^ + 0.6105*P*] where *P* = (*F*_o_^2^ + 2*F*_c_^2^)/3
3997 reflections	(Δ/σ)_max_ < 0.001
325 parameters	Δρ_max_ = 0.27 e Å^−^^3^
0 restraints	Δρ_min_ = −0.25 e Å^−^^3^

### Special details

**Table:**

Geometry. All e.s.d.'s (except the e.s.d. in the dihedral angle between two l.s. planes) are estimated using the full covariance matrix. The cell e.s.d.'s are taken into account individually in the estimation of e.s.d.'s in distances, angles and torsion angles; correlations between e.s.d.'s in cell parameters are only used when they are defined by crystal symmetry. An approximate (isotropic) treatment of cell e.s.d.'s is used for estimating e.s.d.'s involving l.s. planes.
Refinement. Refinement of *F*^2^ against ALL reflections. The weighted *R*-factor *wR* and goodness of fit *S* are based on *F*^2^, conventional *R*-factors *R* are based on *F*, with *F* set to zero for negative *F*^2^. The threshold expression of *F*^2^ > σ(*F*^2^) is used only for calculating *R*-factors(gt) *etc*. and is not relevant to the choice of reflections for refinement. *R*-factors based on *F*^2^ are statistically about twice as large as those based on *F*, and *R*- factors based on ALL data will be even larger.

### Fractional atomic coordinates and isotropic or equivalent isotropic displacement parameters (Å^2^)

**Table:**

	*x*	*y*	*z*	*U*_iso_*/*U*_eq_	
C1	0.3326 (5)	−0.2910 (3)	0.59868 (17)	0.0397 (10)	
C2	0.4198 (5)	−0.2655 (3)	0.54479 (16)	0.0366 (10)	
C3	0.5437 (5)	−0.3374 (4)	0.52782 (18)	0.0544 (12)	
H3	0.5755	−0.4007	0.5507	0.065*	
C4	0.6200 (6)	−0.3150 (4)	0.4769 (2)	0.0653 (14)	
H4	0.7027	−0.3635	0.4653	0.078*	
C5	0.5739 (5)	−0.2205 (4)	0.44316 (19)	0.0583 (13)	
H5	0.6266	−0.2068	0.4089	0.070*	
C6	0.4504 (5)	−0.1452 (3)	0.45896 (16)	0.0384 (10)	
C7	0.3743 (5)	−0.1712 (3)	0.51070 (15)	0.0360 (9)	
H7	0.2906	−0.1235	0.5224	0.043*	
C8	0.3964 (5)	−0.0463 (3)	0.42123 (16)	0.0359 (10)	
C9	0.4790 (5)	−0.0157 (3)	0.37261 (17)	0.0454 (11)	
H9	0.5760	−0.0543	0.3651	0.054*	
C10	0.4217 (5)	0.0706 (3)	0.33468 (17)	0.0445 (11)	
H10	0.4795	0.0887	0.3021	0.053*	
C11	0.2787 (5)	0.1298 (3)	0.34526 (16)	0.0348 (10)	
C12	0.2034 (5)	0.2153 (3)	0.30153 (18)	0.0395 (10)	
C13	0.2002 (5)	0.1057 (3)	0.39553 (17)	0.0455 (11)	
H13	0.1078	0.1481	0.4044	0.055*	
C14	0.2582 (5)	0.0185 (3)	0.43296 (17)	0.0479 (12)	
H14	0.2035	0.0032	0.4666	0.058*	
C15	0.3314 (5)	0.4924 (4)	0.29153 (17)	0.0449 (11)	
H15	0.3656	0.4221	0.3072	0.054*	
C16	0.3820 (5)	0.5911 (4)	0.32141 (17)	0.0499 (12)	
H16	0.4483	0.5865	0.3565	0.060*	
C17	0.3340 (5)	0.6951 (4)	0.29906 (17)	0.0478 (12)	
H17	0.3654	0.7617	0.3191	0.057*	
C18	0.2367 (5)	0.7004 (3)	0.24547 (16)	0.0335 (9)	
C19	0.1832 (5)	0.8018 (3)	0.21576 (19)	0.0428 (11)	
C20	0.1350 (6)	0.9765 (4)	0.1863 (3)	0.0705 (15)	
H20	0.1350	1.0560	0.1855	0.085*	
C21	0.0931 (5)	0.8014 (4)	0.16250 (19)	0.0437 (11)	
C22	0.0414 (5)	0.6983 (3)	0.13349 (17)	0.0388 (10)	
C23	−0.0542 (5)	0.6908 (4)	0.08015 (18)	0.0535 (12)	
H23	−0.0876	0.7567	0.0598	0.064*	
C24	−0.0983 (5)	0.5857 (4)	0.05792 (18)	0.0519 (12)	
H24	−0.1626	0.5793	0.0225	0.062*	
C25	−0.0458 (5)	0.4885 (4)	0.08908 (17)	0.0464 (11)	
H25	−0.0780	0.4174	0.0739	0.056*	
C26	0.0901 (5)	0.5956 (3)	0.16214 (16)	0.0336 (10)	
C27	0.1912 (4)	0.5964 (3)	0.21789 (16)	0.0315 (9)	
Mn1	0.15985 (8)	0.33674 (5)	0.18767 (2)	0.03702 (19)	
N1	0.2089 (4)	0.9153 (3)	0.23044 (17)	0.0551 (10)	
H1	0.2618	0.9414	0.2617	0.066*	
N2	0.0616 (5)	0.9125 (3)	0.14389 (18)	0.0655 (12)	
N3	0.0475 (4)	0.4922 (3)	0.13919 (13)	0.0372 (8)	
N4	0.2358 (4)	0.4944 (3)	0.24136 (13)	0.0358 (8)	
O1	0.3863 (3)	−0.3680 (2)	0.63325 (12)	0.0545 (8)	
O2	0.2030 (4)	−0.2362 (2)	0.60829 (12)	0.0541 (8)	
O3	0.2624 (3)	0.2201 (2)	0.25189 (11)	0.0486 (8)	
O4	−0.0869 (4)	0.2767 (2)	0.18362 (12)	0.0510 (8)	

### Atomic displacement parameters (Å^2^)

**Table:**

	*U*^11^	*U*^22^	*U*^33^	*U*^12^	*U*^13^	*U*^23^
C1	0.045 (3)	0.038 (3)	0.036 (2)	−0.001 (2)	−0.004 (2)	0.005 (2)
C2	0.040 (3)	0.036 (2)	0.033 (2)	−0.002 (2)	−0.003 (2)	0.0031 (19)
C3	0.054 (3)	0.058 (3)	0.052 (3)	0.006 (3)	0.009 (2)	0.017 (2)
C4	0.062 (3)	0.070 (4)	0.067 (3)	0.030 (3)	0.023 (3)	0.020 (3)
C5	0.061 (3)	0.069 (3)	0.046 (3)	0.014 (3)	0.015 (2)	0.022 (3)
C6	0.034 (2)	0.042 (3)	0.039 (2)	−0.002 (2)	−0.0045 (19)	0.000 (2)
C7	0.036 (2)	0.037 (2)	0.034 (2)	0.003 (2)	−0.0036 (18)	0.000 (2)
C8	0.038 (3)	0.037 (2)	0.032 (2)	−0.001 (2)	−0.006 (2)	−0.0001 (18)
C9	0.054 (3)	0.045 (3)	0.039 (2)	0.008 (2)	0.009 (2)	0.007 (2)
C10	0.054 (3)	0.045 (3)	0.036 (2)	0.003 (2)	0.009 (2)	0.003 (2)
C11	0.040 (3)	0.032 (2)	0.031 (2)	0.001 (2)	−0.0053 (19)	0.0021 (17)
C12	0.048 (3)	0.030 (2)	0.040 (3)	−0.006 (2)	0.003 (2)	−0.002 (2)
C13	0.044 (3)	0.042 (3)	0.050 (3)	0.009 (2)	0.007 (2)	0.006 (2)
C14	0.047 (3)	0.054 (3)	0.044 (3)	0.007 (2)	0.011 (2)	0.018 (2)
C15	0.048 (3)	0.047 (3)	0.040 (3)	0.001 (2)	−0.004 (2)	−0.001 (2)
C16	0.054 (3)	0.058 (3)	0.036 (3)	−0.001 (3)	−0.009 (2)	0.000 (2)
C17	0.048 (3)	0.046 (3)	0.048 (3)	−0.009 (2)	0.000 (2)	−0.013 (2)
C18	0.032 (2)	0.029 (2)	0.039 (2)	−0.0037 (19)	0.0022 (19)	−0.0025 (19)
C19	0.044 (3)	0.031 (3)	0.054 (3)	0.002 (2)	0.009 (2)	−0.005 (2)
C20	0.076 (4)	0.032 (3)	0.103 (4)	0.004 (3)	0.005 (3)	0.000 (3)
C21	0.048 (3)	0.033 (3)	0.050 (3)	0.003 (2)	0.006 (2)	0.006 (2)
C22	0.038 (3)	0.038 (3)	0.040 (2)	0.006 (2)	0.005 (2)	0.006 (2)
C23	0.057 (3)	0.052 (3)	0.050 (3)	0.007 (2)	−0.007 (2)	0.012 (2)
C24	0.055 (3)	0.059 (3)	0.039 (3)	0.001 (3)	−0.017 (2)	0.005 (2)
C25	0.057 (3)	0.042 (3)	0.038 (3)	−0.005 (2)	−0.012 (2)	−0.004 (2)
C26	0.032 (2)	0.029 (2)	0.039 (2)	−0.002 (2)	0.0015 (19)	−0.0043 (19)
C27	0.032 (2)	0.030 (2)	0.033 (2)	0.000 (2)	0.0014 (18)	0.0004 (19)
Mn1	0.0488 (4)	0.0288 (3)	0.0331 (3)	−0.0021 (3)	0.0009 (3)	0.0000 (3)
N1	0.063 (3)	0.033 (2)	0.068 (3)	−0.005 (2)	0.002 (2)	−0.011 (2)
N2	0.077 (3)	0.033 (2)	0.084 (3)	0.004 (2)	−0.008 (2)	0.006 (2)
N3	0.041 (2)	0.035 (2)	0.035 (2)	−0.0062 (17)	−0.0028 (16)	0.0010 (16)
N4	0.039 (2)	0.035 (2)	0.0322 (19)	0.0018 (17)	−0.0042 (16)	0.0014 (16)
O1	0.055 (2)	0.061 (2)	0.0474 (18)	0.0062 (16)	0.0023 (15)	0.0234 (16)
O2	0.057 (2)	0.053 (2)	0.0532 (19)	0.0139 (17)	0.0165 (16)	0.0146 (15)
O3	0.064 (2)	0.0449 (18)	0.0371 (16)	0.0085 (15)	0.0060 (15)	0.0079 (14)
O4	0.051 (2)	0.0479 (19)	0.0544 (19)	−0.0143 (16)	0.0055 (15)	−0.0037 (15)

### Geometric parameters (Å, °)

**Table:**

C1—O1	1.253 (4)	C17—H17	0.9300
C1—O2	1.259 (4)	C18—C27	1.406 (5)
C1—C2	1.491 (5)	C18—C19	1.417 (5)
C2—C3	1.384 (5)	C19—C21	1.368 (5)
C2—C7	1.385 (5)	C19—N1	1.382 (5)
C3—C4	1.379 (5)	C20—N2	1.328 (5)
C3—H3	0.9300	C20—N1	1.338 (5)
C4—C5	1.382 (5)	C20—H20	0.9300
C4—H4	0.9300	C21—N2	1.386 (5)
C5—C6	1.398 (5)	C21—C22	1.424 (5)
C5—H5	0.9300	C22—C23	1.393 (5)
C6—C7	1.404 (5)	C22—C26	1.410 (5)
C6—C8	1.487 (5)	C23—C24	1.367 (5)
C7—H7	0.9300	C23—H23	0.9300
C8—C9	1.383 (5)	C24—C25	1.391 (5)
C8—C14	1.391 (5)	C24—H24	0.9300
C9—C10	1.387 (5)	C25—N3	1.321 (4)
C9—H9	0.9300	C25—H25	0.9300
C10—C11	1.382 (5)	C26—N3	1.352 (4)
C10—H10	0.9300	C26—C27	1.458 (5)
C11—C13	1.380 (5)	C27—N4	1.347 (4)
C11—C12	1.508 (5)	Mn1—O4	2.106 (3)
C12—O4^i^	1.250 (4)	Mn1—O3	2.124 (3)
C12—O3	1.262 (4)	Mn1—O2^ii^	2.208 (3)
C13—C14	1.388 (5)	Mn1—N4	2.273 (3)
C13—H13	0.9300	Mn1—N3	2.281 (3)
C14—H14	0.9300	Mn1—O1^ii^	2.313 (3)
C15—N4	1.331 (4)	Mn1—C1^ii^	2.602 (4)
C15—C16	1.387 (5)	N1—H1	0.8600
C15—H15	0.9300	O1—Mn1^iii^	2.313 (3)
C16—C17	1.364 (5)	O2—Mn1^iii^	2.208 (3)
C16—H16	0.9300	O4—C12^i^	1.250 (4)
C17—C18	1.403 (5)		
			
O1—C1—O2	120.6 (4)	N2—C20—N1	113.2 (4)
O1—C1—C2	120.0 (4)	N2—C20—H20	123.4
O2—C1—C2	119.4 (4)	N1—C20—H20	123.4
O1—C1—Mn1^iii^	62.7 (2)	C19—C21—N2	110.0 (4)
O2—C1—Mn1^iii^	57.9 (2)	C19—C21—C22	122.2 (4)
C2—C1—Mn1^iii^	175.7 (3)	N2—C21—C22	127.8 (4)
C3—C2—C7	119.6 (4)	C23—C22—C26	117.9 (4)
C3—C2—C1	120.3 (4)	C23—C22—C21	125.6 (4)
C7—C2—C1	120.1 (4)	C26—C22—C21	116.5 (4)
C4—C3—C2	119.8 (4)	C24—C23—C22	119.5 (4)
C4—C3—H3	120.1	C24—C23—H23	120.3
C2—C3—H3	120.1	C22—C23—H23	120.3
C3—C4—C5	120.2 (4)	C23—C24—C25	119.1 (4)
C3—C4—H4	119.9	C23—C24—H24	120.5
C5—C4—H4	119.9	C25—C24—H24	120.5
C4—C5—C6	121.9 (4)	N3—C25—C24	123.2 (4)
C4—C5—H5	119.1	N3—C25—H25	118.4
C6—C5—H5	119.1	C24—C25—H25	118.4
C5—C6—C7	116.4 (4)	N3—C26—C22	122.0 (3)
C5—C6—C8	121.7 (4)	N3—C26—C27	116.9 (4)
C7—C6—C8	121.8 (4)	C22—C26—C27	121.1 (4)
C2—C7—C6	122.1 (4)	N4—C27—C18	122.5 (3)
C2—C7—H7	119.0	N4—C27—C26	117.1 (3)
C6—C7—H7	119.0	C18—C27—C26	120.4 (4)
C9—C8—C14	117.0 (4)	O4—Mn1—O3	97.67 (11)
C9—C8—C6	121.8 (4)	O4—Mn1—O2^ii^	89.59 (11)
C14—C8—C6	121.2 (4)	O3—Mn1—O2^ii^	98.36 (11)
C8—C9—C10	122.1 (4)	O4—Mn1—N4	120.65 (11)
C8—C9—H9	119.0	O3—Mn1—N4	94.32 (11)
C10—C9—H9	119.0	O2^ii^—Mn1—N4	145.23 (11)
C11—C10—C9	120.0 (4)	O4—Mn1—N3	84.57 (11)
C11—C10—H10	120.0	O3—Mn1—N3	165.21 (11)
C9—C10—H10	120.0	O2^ii^—Mn1—N3	96.26 (11)
C13—C11—C10	118.9 (4)	N4—Mn1—N3	72.22 (11)
C13—C11—C12	119.9 (4)	O4—Mn1—O1^ii^	144.17 (10)
C10—C11—C12	121.1 (4)	O3—Mn1—O1^ii^	100.92 (10)
O4^i^—C12—O3	124.0 (4)	O2^ii^—Mn1—O1^ii^	57.65 (10)
O4^i^—C12—C11	118.4 (4)	N4—Mn1—O1^ii^	88.24 (10)
O3—C12—C11	117.6 (4)	N3—Mn1—O1^ii^	85.10 (11)
C11—C13—C14	120.5 (4)	O4—Mn1—C1^ii^	117.17 (12)
C11—C13—H13	119.8	O3—Mn1—C1^ii^	101.66 (11)
C14—C13—H13	119.8	O2^ii^—Mn1—C1^ii^	28.90 (10)
C13—C14—C8	121.4 (4)	N4—Mn1—C1^ii^	116.67 (12)
C13—C14—H14	119.3	N3—Mn1—C1^ii^	90.13 (11)
C8—C14—H14	119.3	O1^ii^—Mn1—C1^ii^	28.77 (10)
N4—C15—C16	122.5 (4)	C20—N1—C19	106.4 (4)
N4—C15—H15	118.7	C20—N1—H1	126.8
C16—C15—H15	118.7	C19—N1—H1	126.8
C17—C16—C15	119.7 (4)	C20—N2—C21	104.2 (4)
C17—C16—H16	120.2	C25—N3—C26	118.4 (3)
C15—C16—H16	120.2	C25—N3—Mn1	124.9 (3)
C16—C17—C18	119.3 (4)	C26—N3—Mn1	116.5 (2)
C16—C17—H17	120.3	C15—N4—C27	118.5 (3)
C18—C17—H17	120.3	C15—N4—Mn1	124.3 (3)
C17—C18—C27	117.5 (4)	C27—N4—Mn1	116.9 (2)
C17—C18—C19	125.7 (4)	C1—O1—Mn1^iii^	88.5 (3)
C27—C18—C19	116.8 (4)	C1—O2—Mn1^iii^	93.2 (2)
C21—C19—N1	106.2 (4)	C12—O3—Mn1	119.6 (3)
C21—C19—C18	123.0 (4)	C12^i^—O4—Mn1	155.1 (3)
N1—C19—C18	130.9 (4)		
			
O1—C1—C2—C3	10.1 (6)	C22—C26—C27—N4	−178.2 (3)
O2—C1—C2—C3	−168.5 (4)	N3—C26—C27—C18	−178.2 (3)
Mn1^iii^—C1—C2—C3	−118 (4)	C22—C26—C27—C18	2.6 (6)
O1—C1—C2—C7	−171.5 (4)	N2—C20—N1—C19	0.5 (6)
O2—C1—C2—C7	10.0 (6)	C21—C19—N1—C20	−0.1 (5)
Mn1^iii^—C1—C2—C7	60 (4)	C18—C19—N1—C20	178.4 (4)
C7—C2—C3—C4	−0.5 (6)	N1—C20—N2—C21	−0.6 (6)
C1—C2—C3—C4	178.0 (4)	C19—C21—N2—C20	0.5 (5)
C2—C3—C4—C5	0.5 (7)	C22—C21—N2—C20	179.6 (4)
C3—C4—C5—C6	0.1 (7)	C24—C25—N3—C26	1.9 (6)
C4—C5—C6—C7	−0.7 (6)	C24—C25—N3—Mn1	−173.4 (3)
C4—C5—C6—C8	−177.5 (4)	C22—C26—N3—C25	−1.6 (6)
C3—C2—C7—C6	−0.2 (6)	C27—C26—N3—C25	179.2 (3)
C1—C2—C7—C6	−178.6 (3)	C22—C26—N3—Mn1	174.1 (3)
C5—C6—C7—C2	0.7 (5)	C27—C26—N3—Mn1	−5.1 (4)
C8—C6—C7—C2	177.5 (3)	O4—Mn1—N3—C25	−54.9 (3)
C5—C6—C8—C9	−8.1 (6)	O3—Mn1—N3—C25	−154.4 (4)
C7—C6—C8—C9	175.2 (4)	O2^ii^—Mn1—N3—C25	34.1 (3)
C5—C6—C8—C14	170.9 (4)	N4—Mn1—N3—C25	−179.5 (3)
C7—C6—C8—C14	−5.8 (6)	O1^ii^—Mn1—N3—C25	90.8 (3)
C14—C8—C9—C10	−4.2 (6)	C1^ii^—Mn1—N3—C25	62.4 (3)
C6—C8—C9—C10	174.9 (4)	O4—Mn1—N3—C26	129.7 (3)
C8—C9—C10—C11	0.6 (6)	O3—Mn1—N3—C26	30.2 (6)
C9—C10—C11—C13	3.5 (6)	O2^ii^—Mn1—N3—C26	−141.3 (3)
C9—C10—C11—C12	−173.4 (3)	N4—Mn1—N3—C26	5.1 (3)
C13—C11—C12—O4^i^	12.0 (5)	O1^ii^—Mn1—N3—C26	−84.6 (3)
C10—C11—C12—O4^i^	−171.1 (4)	C1^ii^—Mn1—N3—C26	−113.0 (3)
C13—C11—C12—O3	−167.9 (4)	C16—C15—N4—C27	2.2 (6)
C10—C11—C12—O3	9.0 (5)	C16—C15—N4—Mn1	176.6 (3)
C10—C11—C13—C14	−3.9 (6)	C18—C27—N4—C15	−2.4 (6)
C12—C11—C13—C14	173.0 (4)	C26—C27—N4—C15	178.4 (3)
C11—C13—C14—C8	0.3 (6)	C18—C27—N4—Mn1	−177.2 (3)
C9—C8—C14—C13	3.7 (6)	C26—C27—N4—Mn1	3.6 (4)
C6—C8—C14—C13	−175.3 (4)	O4—Mn1—N4—C15	108.8 (3)
N4—C15—C16—C17	−0.4 (7)	O3—Mn1—N4—C15	7.2 (3)
C15—C16—C17—C18	−1.3 (6)	O2^ii^—Mn1—N4—C15	−104.2 (3)
C16—C17—C18—C27	1.1 (6)	N3—Mn1—N4—C15	−179.0 (3)
C16—C17—C18—C19	−177.3 (4)	O1^ii^—Mn1—N4—C15	−93.6 (3)
C17—C18—C19—C21	177.7 (4)	C1^ii^—Mn1—N4—C15	−98.1 (3)
C27—C18—C19—C21	−0.7 (6)	O4—Mn1—N4—C27	−76.8 (3)
C17—C18—C19—N1	−0.6 (7)	O3—Mn1—N4—C27	−178.3 (3)
C27—C18—C19—N1	−179.0 (4)	O2^ii^—Mn1—N4—C27	70.3 (3)
N1—C19—C21—N2	−0.2 (5)	N3—Mn1—N4—C27	−4.6 (3)
C18—C19—C21—N2	−178.9 (4)	O1^ii^—Mn1—N4—C27	80.9 (3)
N1—C19—C21—C22	−179.4 (4)	C1^ii^—Mn1—N4—C27	76.3 (3)
C18—C19—C21—C22	2.0 (7)	O2—C1—O1—Mn1^iii^	2.3 (4)
C19—C21—C22—C23	178.2 (4)	C2—C1—O1—Mn1^iii^	−176.2 (3)
N2—C21—C22—C23	−0.8 (7)	O1—C1—O2—Mn1^iii^	−2.4 (4)
C19—C21—C22—C26	−0.9 (6)	C2—C1—O2—Mn1^iii^	176.1 (3)
N2—C21—C22—C26	−179.9 (4)	O4^i^—C12—O3—Mn1	−0.8 (5)
C26—C22—C23—C24	0.6 (6)	C11—C12—O3—Mn1	179.1 (2)
C21—C22—C23—C24	−178.4 (4)	O4—Mn1—O3—C12	−57.7 (3)
C22—C23—C24—C25	−0.3 (7)	O2^ii^—Mn1—O3—C12	−148.5 (3)
C23—C24—C25—N3	−1.0 (7)	N4—Mn1—O3—C12	64.0 (3)
C23—C22—C26—N3	0.4 (6)	N3—Mn1—O3—C12	40.1 (6)
C21—C22—C26—N3	179.5 (3)	O1^ii^—Mn1—O3—C12	153.0 (3)
C23—C22—C26—C27	179.6 (4)	C1^ii^—Mn1—O3—C12	−177.6 (3)
C21—C22—C26—C27	−1.3 (6)	O3—Mn1—O4—C12^i^	4.0 (7)
C17—C18—C27—N4	0.8 (6)	O2^ii^—Mn1—O4—C12^i^	102.4 (6)
C19—C18—C27—N4	179.3 (3)	N4—Mn1—O4—C12^i^	−95.7 (7)
C17—C18—C27—C26	179.9 (3)	N3—Mn1—O4—C12^i^	−161.3 (7)
C19—C18—C27—C26	−1.5 (5)	O1^ii^—Mn1—O4—C12^i^	124.8 (6)
N3—C26—C27—N4	1.0 (5)	C1^ii^—Mn1—O4—C12^i^	111.3 (6)

Symmetry codes: (i) −*x*, *y*, −*z*+1/2; (ii) *x*, −*y*, *z*−1/2; (iii) *x*, −*y*, *z*+1/2.
